# Does cognition affect supervised and unsupervised mobility differently in people with Parkinson’s disease? A cross-sectional study

**DOI:** 10.1186/s12984-025-01873-9

**Published:** 2026-01-24

**Authors:** Edoardo Bianchini, Francesco Garramone, Domiziana Rinaldi, Marika Alborghetti, Lanfranco De Carolis, Silvia Galli, Antonio Suppa, Marco Salvetti, Clint Hansen, Nicolas Vuillerme

**Affiliations:** 1 Department of Neuroscience, Mental Health and Sensory Organs (NESMOS), Sapienza University of Rome, Via di Grottarossa 1035, 00189 Rome, Italy; 2 Univ. Grenoble Alpes, CNRS, Grenoble INP, LIG Sangria, 38000 Grenoble, France; 3 Department of Human Neurosciences, Sapienza University of Rome, 00185 Rome, Italy; 4 IRCCS Neuromed Institute, 86077 Pozzilli, Italy; 5 Department of Neurology, Kiel University, 24105 Kiel, Germany; 6 Institut Universitaire de France, 75005 Paris, France

**Keywords:** Mobility, Cognition, Parkinson’s disease, Gait, Smartwatch, Daily steps, Digital outcome measures, Supervised mobility, Unsupervised mobility

## Abstract

**Background:**

The co-existence of cognitive and mobility impairments is frequent in people with Parkinson’s disease (PwPD). Cognitive functions are associated with different types of supervised mobility, but much less is known about unsupervised mobility.

**Methods:**

Mild-to-moderate PwPD, without severe cognitive impairment and not using walking aids, performed three hospital-based supervised mobility tasks: forward walking, instrumented timed-up-and-go (iTUG) with a lower-back-mounted inertial sensor and a 3-meter backward walking test. Forward walking speed (FWS), backward walking speed (BWS) and mean angular velocity (MAV) of the 180° turn during iTUG were calculated. Unsupervised mobility was monitored through average daily steps (AvDS) measured over 5 days by means of a commercial smartwatch (Garmin Vivosmart 4) in free-living conditions. Cognitive performance was evaluated using the Montreal Cognitive Assessment (MoCA) and the Word-Colour Stroop Test (WCST). Multivariable linear regression models with backward elimination method were used to evaluate how much variance of each mobility measure was explained by cognitive functions and the effect of other clinical and demographic variables.

**Results:**

A total of 105 PwPD were recruited. Regarding supervised mobility, multivariable regressions revealed that cognitive functions explained 12.3% (*P* < 0.001), 12.4% (*P* < 0.001) and 15.0% (*P* < 0.001) variance of FWS, MAV and BWS, respectively. For unsupervised mobility, cognitive functions explained only 5.5% (*P* = 0.009) of AvDS variance. WCST was included in all regression models and was a significant predictor of all mobility parameters, either supervised or unsupervised. MoCA was included only in the model for supervised BWS and was not a significant predictor. When considering clinical and demographic variables, neither MoCA nor WCST survived as significant predictors.

**Conclusions:**

Our findings support the role of executive functions in PwPD mobility. Cognition correlated with supervised mobility more than unsupervised, likely due to the role of individual and environmental factors. These findings suggest that a comprehensive approach to PD should include cognitive assessments alongside traditional motor evaluation. Mobility evaluation in PwPD should be performed in both supervised and unsupervised conditions.

## Highlights

Cognition, particularly executive functions, correlates with supervised more strongly than with unsupervised mobility in people with Parkinson’s disease (PD).

In supervised conditions, cognitive functions are associated with backward walking more strongly than with forward walking and turning.

When clinical and demographic variables are included, cognitive predictors lose statistical significance.

Motor-cognitive evaluation should be integrated in PD clinical assessment and management.

Mobility in PD should be assessed under both supervised and unsupervised conditions.

## Introduction

Parkinson’s disease (PD) is a progressive neurodegenerative disorder that primarily manifests with motor symptoms such as bradykinesia, rigidity, and tremor [1]. Among motor manifestations of the disease, impaired gait and functional mobility are not uncommon, particularly with disease progression, and have a profound impact on quality of life [2]. Gait and functional mobility can be measured through various standardized tests in the clinical setting. However, a growing body of evidence showed that what can be seen during clinical evaluation does not closely reflects real-world mobility. Indeed, in-hospital mobility parameters of people with PD (PwPD) correlate only partially with those obtained in real-world setting environment [3–5]. Assessments in supervised setting are usually triggered by a command and done in an isolated, standardized setting with limited ecological validity. In response to these challenges, during recent years it has been a growing interest in mobile health technologies for evaluating unsupervised mobility of people with movement disorders, including PD [6]. Indeed, inertial motion units integrated into these devices allow the collection of various mobility parameters, such as daily step count in free-living, unsupervised conditions. Although this parameter does not provide detailed insights of subtle gait disorders, a reduced number of daily steps is associated with risk of overall mortality [7] and various health conditions such as cancer, cardiovascular diseases, and dementia [8, 9]. Previous studies have also demonstrated an inverse correlation between the number of daily steps and disease severity in PwPD [10, 11].

Besides classical PD motor manifestations, non-motor symptoms are increasingly recognized as key features of the disease. Among these, cognitive dysfunction significantly impact quality of life in PwPD [12]. It encompasses deficits across various domains, including executive functions, attention, memory, and visuospatial abilities [13]. Previous evidence reported an association between cognition, in particular executive and visuospatial functions, and different types of supervised gait and mobility tasks such as forward and backward walking and turning [14–18]. Indeed, mobility impairments in PD are not merely a consequence of motor symptoms and are closely linked to cognitive decline [19, 20]. Executive functions (involving planning, decision-making, and task-switching abilities) play a crucial role in coordination and execution of complex motor tasks such as walking, especially under dual-task conditions and when more complex forms of gait are performed (e.g., turning, sideways and backward walking, etc.) [21–23]. Previous studies have shown that PwPD who experience difficulties in executive functions are more prone to gait disturbances, such as freezing of gait (FOG) [24], and are at a higher risk of falls [25]. This relationship suggests an interplay between cognitive and motor dysfunctions in PD, potentially sharing underlying neuropathological mechanisms. However, the association between cognitive functions and mobility was explored in a supervised setting [21–23] and very few evidence are available on unsupervised mobility [24].

To our knowledge no studies have investigated the contribution of cognition to both supervised and unsupervised mobility in PwPD. Indeed, previous studies have evaluated the association between cognitive function and each mobility state separately. Therefore, this study aimed to investigate the association between cognitive functions and both supervised and unsupervised mobility and the contribution of cognition to mobility in PwPD.

## Materials and methods

### Ethics

Approval was granted by the local Ethical Committee of Sapienza, University of Rome (Ref. 0372/2022). This study was conducted in accordance with the ethical standards set forth in the Declaration of Helsinki of 1964 and subsequent amendments. Data collection and processing were carried out in compliance with current European data protection regulations.

### Population

Participants were consecutively recruited from the Movement Disorders Centre at Sant’Andrea University Hospital (A.O.U. Sant’Andrea) in Rome between February 2023 and May 2024, in accordance to the following inclusion criteria: (i) diagnosis of idiopathic Parkinson’s disease according to the Movement Disorders Society (MDS) criteria [26]; (ii) age 18 years or older; (iii) disease stage < 4 according to the modified Hoeh and Yahr scale (mHY) (“severe disability; still able to walk or stand unassisted”) [27]; (iv) ability to perform the experimental procedure. Exclusion criteria were defined as: (i) Montreal Cognitive Assessment (MoCA) score < 18 [28]; (ii) orthopaedic, rheumatologic, or systemic conditions affecting mobility as judged by the assessor.

### Experimental procedure

This was a cross-sectional study aimed at investigating the association between cognitive functions, and both supervised and unsupervised mobility in PwPD. Participants meeting the inclusion and exclusion criteria were evaluated during their regular follow-up visits at the Neurology clinics of A.O.U. Sant’Andrea in Rome.

Anthropometric (weight, height, BMI) and clinical-demographic data (age, sex, disease duration, levodopa equivalent daily dose [LEDD], mHY) were collected. Participants were evaluated through the following clinical scales: Movement Disorder Society Unified Parkinson’s Disease Rating Scale (MDS-UPDRS) part III (MDS-UPDRS-III) [29] was used to assess motor symptoms severity, Montreal cognitive assessment (MoCA) [30] was used to evaluate global cognition. The time to complete the interference part of the word-colour Stroop test (WCST) in seconds was used to evaluate executive functions [31].

Subsequently, participants were invited to perform two supervised mobility tasks while wearing a lower-back mounted research-grade inertial device for mobility analysis (BTS G-WALK, BTS Engineering Inc., Milan) [32]: (i) a 20-meters Walking Test; and (ii) an instrumented Timed Up and Go (TUG) [33]. From these tasks, the following variables were computed in order to facilitate the comparison between the 2 supervised mobility tasks: forward walking speed (m/s) (FWS); mean angular velocity (°/s) (MAV) of the intermediate 180-degree turn during the TUG. Additionally, a 3-meter Backward Walk Test (3MBWT) was performed according to previous validation studies [34, 35]. PwPD were instructed to walk backward along a 3-meter walkway as quickly as possible while maintaining safety. An assessor followed each participant to ensure safety throughout the task. The test was repeated three times, and the completion time for each trial was recorded using a stopwatch. Backward walking speed (m/s)(BWS) was then calculated by dividing the distance (3 m) by the completion time, to obtain a speed rather than a time metric and harmonize it with the other speed/velocity supervised mobility parameters. The average of the three trials was used for analysis. Garmin Vivosmart 4 smartwatch (Garmin, Olathe, KS, USA, configured according to the manufacturer’s specifications) by indicating the users’ age, height, weight, was then worn on the wrist of the less affected side (i.e., left or right) [36] for 5 consecutive days, in line with previous findings showing that a minimum of 4 days of monitoring is needed to reliably estimate daily step count in PwPD [37]. Participants were instructed to wear the smartwatch all time during day and night and remove it only when involved in water activities (e.g., bathing, showering, swimming, etc.). Participants were also asked to perform daily activities as usual. No additional reminder or instructions were provided to participants. During the monitoring period, participants could access information on the smartwatch screen, including the total number of steps, heart rate and sleep time. The device did not provide instructions, alerts or other messages to participants.

**Fig. 1:**
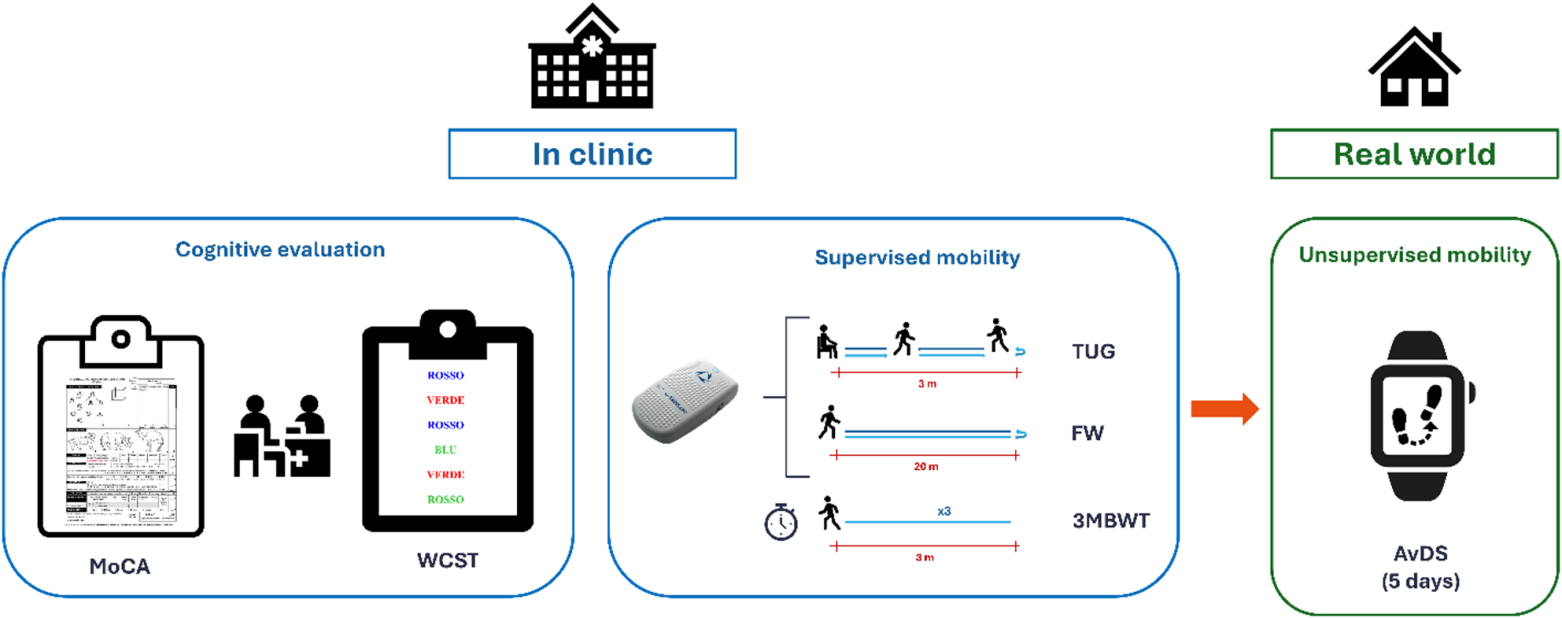
Schematic representation of study experimental procedure. *3MBWT* 3-meter backward walking test; *AvDS* average daily steps, *FW* forward walking, *MoCA* Montreal Cognitive Assessment, *TUG* Timed Up and Go, *WCST* Word-Colour Stroop test

The total daily steps recorded were extracted, and the average daily steps (AvDS) were computed [37]. Compliance was assessed based on the participants’ dashboard data. We could assess that the device was worn through heart rate and activity data. We considered all recording days with ≥ 80% wear time while awake to be valid. We estimated the awake time as 24 h minus the total sleep time as indicated by the device. Experimental procedure is summarized in Fig. 1.

### Statistical analyses

Statistical analyses were performed using JASP v0.18.3.0 (JASP Team, University of Amsterdam, The Netherlands), R v4.0.3, and R Studio v2023.12.0 + 369 for Windows (R Foundation for Statistical Computing, Vienna, Austria). Descriptive statistics were calculated for the considered variables, and the normality was assessed by inspecting histograms and residual plots. Pearson’s test was used to evaluate correlations between cognitive test scores and supervised and unsupervised mobility parameters. The cut-off points used for interpreting correlation coefficient values were as follows [38]: < 0.1: negligible; 0.1–0.2: very weak; 0.2–0.4: weak; 0.4–0.7: moderate; 0.7–0.9: strong; > 0.9: very strong. Subsequently, to evaluate how much variance in supervised and unsupervised mobility parameters was explained by participants’ cognitive performance, four separate multivariable linear regression models were constructed, with mobility-related variables as dependent variables and cognitive test scores as independent variables. A backward elimination method was used to determine which independent variables were significant predictors of the model [39], with the elimination threshold set at *p* ≥ 0.1. The adjusted R² value was calculated for each resulting model. Finally, we performed four additional multivariable analyses with backward elimination, including age, sex, disease duration, LEDD, and MDS-UPDRS III to account for other potentially relevant predictors of mobility in PD. For each model, the presence of influential points was assessed using Cook’s distance, with values > 1 considered influential. For all analyses, the significance threshold was set at alpha < 0.05. All data are reported as mean ± SD or median (Q1-Q3) for numerical variables and N (%) for categorical variables.

## Results

A total of 105 PwPD who met the pre-specified inclusion and exclusion criteria were enrolled in the study. All participants were monitored through Garmin Vivosmart 4 at home for a period of 5 consecutive days. No participants or days were excluded based on the prespecified compliance criteria. The demographic, anthropometric, clinical and mobility features in supervised and unsupervised conditions are summarized in Table 1.

**Table 1:** Demographic, anthropometric, clinical and mobility features of the study population

	PwPD (*N* = 105)
Age (years)	68.2 ± 8.7
Gender (female)	34 (32%)
Height (cm)	171 ± 8.7
Weight (kg)	76.0 ± 13.2
BMI	25.8 ± 3.4
Disease duration (years)	5.6 ± 3.8
LEDD (mg)	501 ± 237
mHY	2 (2-2.5)
MDS-UPDRS-III	26 (21–32)
MoCA	26 (25–28)
WCST (s)	41.6 ± 16.1
Supervised mobility parameters	
FWS (m/s)	1.19 ± 0.22
BWS (m/s)	0.60 ± 0.22
MAV (deg/s)	70.9 ± 17.9
Unsupervised mobility parameters	
AvDS (steps/day)	5979 ± 3037

*AvDS* average daily steps, *BWS* backward walking speed, *FWS,* forward walking speed, *LEDD* levodopa equivalent daily dose, *MAV* mean angular velocity, *MDS-UPDRS-III* Movement Disorder Society Unified Parkinson’s Disease Rating Scale part III, *mHY* modified Hoehn and Yahr scale, *MoCA* Montreal Cognitive Assessment, *WCST* Word-Colour Stroop test

### Correlations

#### Supervised mobility

Pearson’s correlation test revealed a weak negative correlation between the WCST completion time and FWS (*R* = -0.362; *p* < 0.001), MAV (*R* = -0.364; *p* < 0.001), and BWS (*R* = -0.379; *p* < 0.001).

There was also a weak positive correlation between the MoCA score and FWS (*R* = 0.316; *p* = 0.001), MAV (*R* = 0.306; *p* = 0.002), and BWS (*R* = 0.328; *p* < 0.001).

#### Unsupervised mobility

Pearson’s correlation test revealed a weak negative correlation between the WCST completion time and AvDS (*R* = -0.25; *p* = 0.009). There was no significant correlation between the MoCA score and AvDS (*R* = 0.114; *p* = 0.25).

**Fig. 2:**
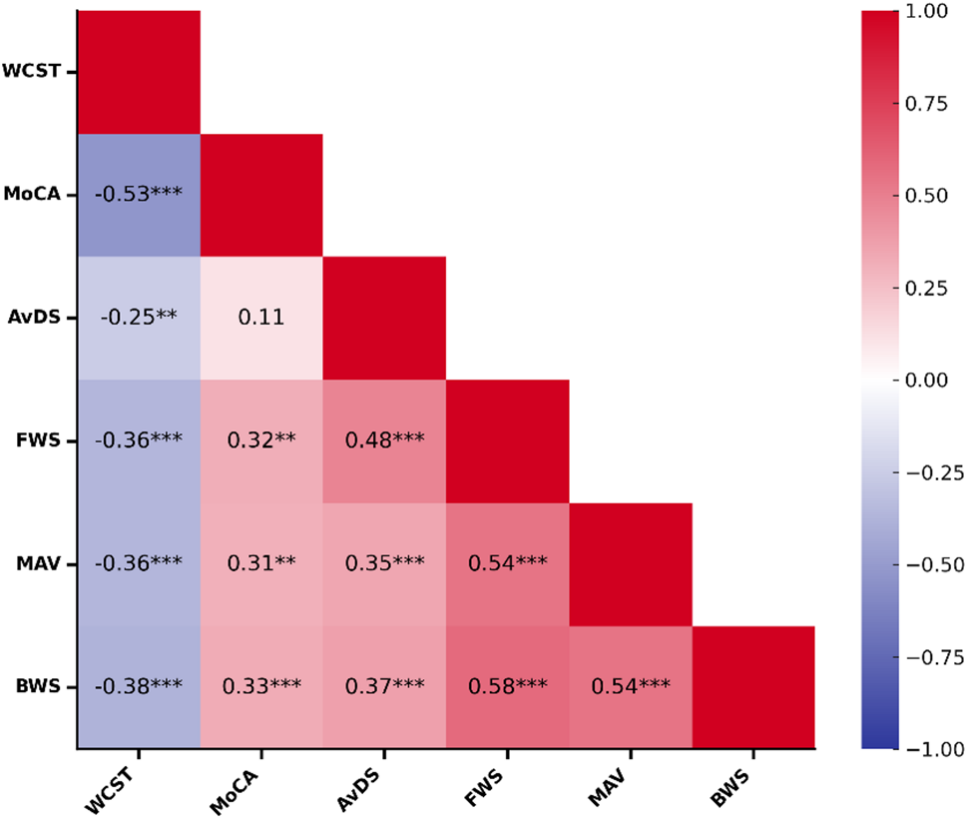
Heatmap showing correlations between variables. A colour gradient was used to indicate the strength of positive (red) and negative (blue) associations. Pearson’s correlation coefficient for each pair of variables is reported in each square. Significance of correlation is marked with asterisk as follows: * = *P* < 0.05; ** = *P* < 0.01; *** = *P* < 0.001. *AvDS* average daily steps, *BWS* backward walking speed, *FWS* forward walking speed, *MAV* mean angular velocity, *MoCA* Montreal Cognitive Assessment, *WCST* Word-Colour Stroop test

Correlations are summarized in Fig. 2.

### Linear regression analysis

#### Supervised mobility

**Fig. 3:**
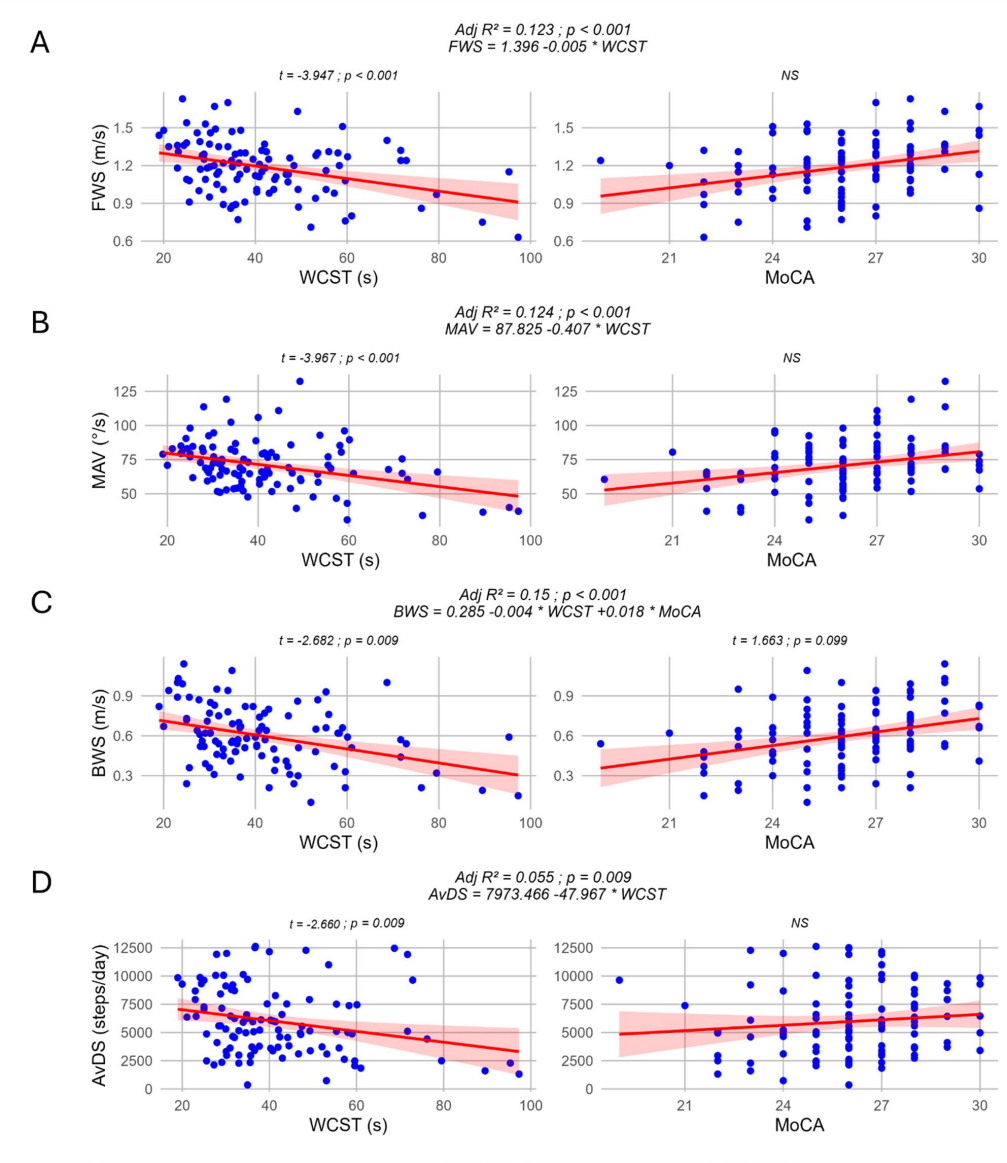
Linear regression models and scatterplot of paired independent and dependent variables. Panel **A**: Linear regression model for FWS. Panel **B**: Linear regression model for MAV. Panel **C**: Linear regression model for BWS. Panel **D**: Linear regression model for AvDS. For each graph, a linear regression line was added. The shaded area indicates the 95% confidence interval. Adjusted R^2^, p-values and regression equations of each model after backward elimination were added above each variable pair. T-statistics and p-value was also added on top of each significant predictor. NS was added for non-significant predictors. *AvDS* average daily steps, *BWS* backward walking speed, *FWS* forward walking speed, *MAV* mean angular velocity, *MoCA* Montreal Cognitive Assessment, *WCST* Word-Colour Stroop test

For FWS, the linear regression model following backward elimination method, identified only the WCST completion time as a statistically significant predictor (t = -3.947; *p* < 0.001). The final regression model was statistically significant (F = 15.581; adjR² = 0.123; *p* < 0.001) (Fig. 3A).

For MAV during the TUG, the linear regression model also identified the WCST completion time as the only statistically significant predictor after backward elimination (t = -3.967; *p* < 0.001). The final regression model was statistically significant (F = 15.738; adjR² = 0.124; *p* < 0.001) (Fig. 3B).

For BWS, the linear regression model included both WCST completion time (t = -2.682; *p* = 0.009) and MoCA score (t = 1.663; *p* = 0.099) as predictors for the overall model but only WCST completion time reached the significance. The final regression model was statistically significant (F = 10.168; adjR² = 0.150; *p* < 0.001) (Fig. 3C). Since the p-value of MoCA in the final model was very close to the threshold for inclusion, we also assessed the model without this variable. This model was statistically significant (F = 17.273; R² = 0.144; *p* < 0.001; WCST t = -4.156; *p* < 0.001). No influential points (Cook’s distance > 1) were found in the models.

#### Unsupervised mobility

For AvDS, the linear regression model, after backward elimination, identified only the WCST completion time as a statistically significant predictor (t = -2.660; *p* = 0.009). The final regression model was statistically significant (F = 7.077; R² = 0.055; *p* = 0.009) (Fig. 3D). No influential points (Cook’s distance > 1) were found in the models.

### Multivariable analysis including clinical and demographical variables

For FWS, age, sex, MDS-UPDRS-III and LEDD were included in the regression model and all variables but LEDD were significant predictors. For MAV, WCST, MDS-UPDRS-III, sex, MoCA and LEDD were included in the regression model and only MDS-UPDRS-III was a significant predictor. For BWS, age, disease duration, MDS-UPDRS-III, sex and LEDD were included in the regression model and were a significant predictor. Finally, for AvDS, age, MDS-UPDRS-III, sex and LEDD were included in the regression model and all variables but sex were significant predictors. No influential points (Cook’s distance > 1) were found in the models. Details of the multivariable regression models are shown in Table 2.

**Table 2:** Details of the multivariable regression models showing overall significance, adjusted R²

Variable	F-statistics	aR² (%)	*p*-value (model)	Predictors	Coefficients	*p*-value (coeff.)
FWS	13.628	32.7	< 0.001	(Intercept)	12.718	< 0.001***
				Age	-2.958	0.004**
				MDS-UPDRS III	-3.329	0.001**
				Sex (M)	2.845	0.005**
				LEDD	-1.731	0.087
MAV	6.968	22.3	< 0.001	(intercept)	2.787	0.006**
				WCST	-1.844	0.068
				MDS-UPDRS III	-2.371	0.02*
				Sex (M)	1.029	0.306
				MoCA	1.088	0.279
				LEDD	-1.731	0.087
BWS	18.590	45.8	< 0.001	(Intercept)	11.522	< 0.001***
				Age	-5.448	< 0.001***
				Disease duration	2.184	0.031*
				MDS-UPDRS III	-3.306	0.001**
				Sex (M)	3.742	< 0.001***
				LEDD	-3.072	0.003**
AvDS	11.893	29.5	< 0.001	(Intercept)	7.437	< 0.001***
				Age	-2.652	0.009**
				MDS-UPDRS III	-3.136	0.002**
				Sex (M)	1.574	0.119
				LEDD	-2.315	0.023*

Predictors included in the model after backward elimination and their coefficients and p-values

*AvDS* average daily steps, *BWS* backward walking speed, *FWS* forward walking speed, *LEDD* levodopa equivalent daily dose, *MAV* mean angular velocity, *MDS-UPDRS-III* Movement Disorder Society Unified Parkinson’s Disease Rating Scale part III, *MoCA* Montreal Cognitive Assessment, *WCST* Word-Colour Stroop test

## Discussion

This study aimed to explore the association between cognitive functions and both supervised and unsupervised mobility and the contribution of cognition to mobility in PwPD. Our findings revealed significant associations between cognition, particularly executive functions, and mobility in supervised and unsupervised conditions, emphasizing the role of cognitive processes in maintaining mobility in PwPD. However, when considering other clinical and demographics metrics, cognitive predictors lost their significance.

### Cognitive functions and supervised mobility 

Our unadjusted models indicate that executive functions, as measured by the WCST, may be a significant predictor of supervised mobility. Specifically, FWS, BWS and MAV were significantly associated with WCST performance. The WCST requires the prefrontal cortex to suppress automatic responses and engage in cognitive flexibility [31]. The frontal cortex and, particularly, the prefrontal areas, are heavily involved in planning, initiation, and execution of complex motor tasks, including walking [40]. Imaging studies have shown activation of these areas during gait, supporting the notion that executive dysfunction can directly impact motor performance [22, 41, 42]. Impairments in these executive functions can lead to difficulties in adapting to changing environmental demands, which is essential for safe and efficient mobility.

Our finding aligns with existing literature that highlights the critical role of frontal-executive functions in gait and motor control in both healthy elderly and PwPD [14, 15]. Previous studies in PwPD, indeed, highlighted how executive functions are linked to deterioration in spatiotemporal gait parameters, in particular regarding the domains of pace and rhythm [19, 20]. Furthermore, executive functions have been associated with different types of mobility such as turning [22] and backward walking [43]. The association between gait and cognitive dysfunction could point toward a shared neurodegenerative basis. In particular, the basal ganglia are implicated in gait control and cognitive processes, particularly those related to executive functions. Thus, the degeneration of dopaminergic neurons in the substantia nigra, could disrupt the circuitry between basal ganglia and prefrontal cortex, leading to both motor and cognitive deficits [43]. This disruption could contribute to difficulties in initiating and maintaining gait in PwPD, particularly under conditions requiring cognitive engagement, such as navigating obstacles or multitasking [44].

Furthermore, the interaction between cognitive decline and mobility in PD could be influenced by non-dopaminergic systems such as cholinergic [45], noradrenergic [46], GABA and glutamatergic neurotransmission dysfunction [47]. The complexity of these interactions underscores the need for comprehensive management strategies that address both cognitive and motor aspects of the disease.

Interestingly, in our study, global cognitive functions, as assessed by the MoCA, were included in the linear regression model only for BWS test, although with minimal contribution, and not for FWS or MAV and a slightly higher portion of variance was explained by cognition for BWS compared to FWS and MAV (15% vs. 12.3% vs. 12.4%, respectively). This result may suggest that backward walking, a more complex and less practiced motor task, places greater cognitive demands on PwPD [48]. The increased difficulty of the task, which lacks visual cues and requires higher levels of proprioceptive integration and motor planning, could explain the stronger association with global cognitive function [18]. This finding underscores the importance of including varied mobility tasks in clinical assessments to capture the full spectrum of motor-cognitive interactions in PD.

### Cognitive functions and unsupervised mobility

Differently from supervised mobility, unsupervised mobility, as measured by AvDS, even though associated with executive functions, displayed a weaker correlation with it. In fact, WCST explained only a modest proportion of the variance (5.5%) of AvDS. This might depend on the mobility measure adopted. Indeed, a very recent study by Burgos and colleagues [49] reported that executive and visuospatial domains of cognition are associated with spatiotemporal gait parameters such as gait speed, stride length, and double support time in real-world conditions. Daily step count represents a global measure of mobility and physical activity, providing an estimate of how much an individual walks on average throughout the day. However, this measure does not capture subtle and detailed gait features or parameters. The development of validated real-world gait measures is currently a hot research topic, and several international consortia are actively working toward this goal [50]. To date, however, these efforts rely mainly on research-grade devices, thus limiting the advantages of commercial wearables in terms of scalability, accessibility, and ease of use. Nevertheless, previous studies underlined the association between daily steps and cognition in older adults [51] and in different populations of people with neuropsychiatric disorders [52, 53]. Our findings are in line with those of Mc Ardle and colleagues [24], reporting that frontal executive functions, as measured by the verbal fluency task, were a significant predictor of daily step count, measured by a research-grade, lower-back mounted, triaxial accelerometer for 7 days, in a population of 64 mild-to-moderate PwPD. We validate these findings in a larger PwPD population (*n* = 105), using a consumer-grade instrument to collect AvDS. Moreover, we recently reported that the minimal clinically important difference (MCID) for AvDS, measured with the Garmin Vivosmart 4 in PwPD, was 1200 steps/day for clinical-related measures [54]. Based on the present regression results, each 1-second change in the WCST interference part corresponds to a variation of 48 steps. Thus, a change of approximately 25 s would be required to reach a clinically meaningful difference in AvDS. Notably, this value falls within the reported MCID range for WCST interference in older adults at risk of falls (11.5 to 26.0 s) [55], which provides additional clinical relevance to our findings.

Nevertheless, the weaker association between cognition and unsupervised mobility could also reflect the influence of environmental, contextual and individual factors that affect free-living movements but are not captured in a controlled laboratory setting [56]. Indeed, previous evidence highlighted the discrepancy between capacity (what an individual can do) and performance (what an individual does do) [3–5]. In the real world, mobility is often influenced by a multitude of factors beyond cognitive ability, such as environmental barriers, weather, work and socio-economic status, motivation, pain, fatigue, and physical health conditions [57]. Thus, while cognitive functions are crucial for specific, controlled motor tasks, their influence on everyday mobility might be modulated and dampened by other external factors.

### Multivariable analysis including clinical and demographical variables

We found that after including clinical and demographic covariates (age, sex, disease duration, LEDD, and MDS-UPDRS III), WCST and MoCA did not survive as significant predictors in the regression models for FWS, BWS, and AvDS after backward elimination. Cognitive variables survived in the regression model for MAV after including clinical and demographic variables but did not emerge as significant predictors. The predominant effect of age is expected, given its well-established contribution to mobility and gait, with numerous studies demonstrating the impact of ageing on walking in both healthy adults [58] and PwPD [59]. This is also consistent with the recognized predictive value of gait speed for mortality in older adults [60] and in healthy ageing in general [61]. The strong and consistent predictive role of the MDS-UPDRS part III is likewise unsurprising, as gait and mobility are motor functions, and this aligns with previous findings [2, 62, 63]. Similarly, the impact of disease duration is in line with its close association with neurodegeneration [64] and disease severity [65], in this progressive disorder. The effect of LEDD also reflects prior evidence showing its influence on gait parameters deterioration over time [66]. While dopaminergic therapy remains the cornerstone of PD management [1], the response to treatment of gait and mobility disturbances, as well as other axial symptoms, may hinder in advanced stages [67]. Nevertheless, walking parameters often retain some responsiveness to pharmacological treatment [2]. Finally, sex differences in mobility outcomes have been reported in PwPD [68, 69]. This may explain why sex was a significant predictor of FWS and BWS in our models. In summary, our multivariable analysis indicates that although global and executive functions can correlate with mobility in PwPD, their influence is outweighed by stronger determinants such as clinical and demographic variables.

### Implications for clinical practice

The association between executive dysfunction and impaired supervised mobility underscores the importance of incorporating cognitive rehabilitation strategies into PD management. Interventions targeting inhibitory control and selective attention may have the potential to improve motor performance and reduce risk of falls [70, 71]. Previous studies underlined the positive effect of training cognitive and motor functions together (motor-cognitive dual-task) on both supervised gait and cognitive abilities in PwPD [72–74]. Moreover, previous evidence demonstrated that cognitive training alone could lead to an improvement in motor symptoms [70] and vice versa [75]. Moreover, the significant association between global cognitive function and BWS emphasizes the value of including diverse mobility assessments in clinical evaluations. Backward walking tests might indeed serve as an indicator of both motor and cognitive impairments, facilitating early identification of people at higher risk of mobility decline [18, 76]. Additionally, Shen and Mak [77] reported that step training including backward training improved balance and mobility in PwPD, supporting the inclusion of such tasks in rehabilitation programs. Nevertheless, the modest correlation between cognitive function and unsupervised mobility, as well as the predominant role of clinical and demographic parameters in the multivariable analysis, highlight the need for comprehensive, multidimensional assessments that consider both cognitive, clinical, individual and environmental factors when planning interventions for PwPD.

### Limitations and future directions

Despite the novel insights provided by this study, several limitations must be acknowledged. First, the cross-sectional design limits our ability to infer causality between cognitive functions and mobility outcomes. Longitudinal studies are needed to determine whether cognitive decline precedes or follows mobility impairments in PD and clarify this association. Additionally, while we focused on executive functions, other cognitive domains, such as visuospatial abilities, might also play a critical role in mobility [15] and are commonly altered in PwPD [13]. These should be explored in future research. We also included only PwPD with MoCA ≥ 18. While this is a lower threshold, compared with other proposed MoCA cut-offs reported in literature [78, 79], yet we did not investigate the entire spectrum of cognitive impairment in PD. Therefore, we could not draw information regarding the relationship between mobility and cognition in PwPD with severe cognitive impairment. Indeed, it is likely that in more advanced stages of the disease, severe cognitive deficits and dementia could have a detrimental effect on mobility, as reported previously [80]. However, we decided to focus on the PD population with sufficiently preserved cognitive functions and most likely to use consumer-grade wearable devices.

We only considered AvDS as unsupervised mobility parameter, in line with previous research [3]. A growing body of evidence highlighted the possibility of collecting, measuring and monitoring gait parameters in free-living real world settings [81, 82] and several international consortia are working on validating and refining this possibility [50, 83]. Future studies are warranted to include more detailed analysis of real-world mobility in order to more accurately assess the association with cognition.

Furthermore, the use of consumer-grade smartwatch for unsupervised mobility data collection may have reduced the accuracy and reliability in AvDS collection, particularly in people with more severe PD or those exhibiting tremor and dyskinesias. However, previous studies from ours and other groups highlighted the accuracy and reliability of AvDS estimation in mild-to-moderate PwPD [36, 37, 84]. In addition, because the device algorithm is not publicly available, we could not assess its potential impact on our results. For example, the Garmin Vivosmart 4 requires users to input age, weight, and height into their profile, and we followed these instructions to ensure that smartwatch use reflected typical real-world conditions. However, whether and how the device incorporates this information into its calculations remains unknown, as the algorithm is proprietary. Nevertheless, the use of commercially available device could facilitate the capillarity collection of mobility measures in clinical practice, thus providing valuable insight in PwPD conditions in free-living, real-world settings.

The possibility for participants to access their smartwatch data may have influenced their physical activity levels during the monitoring period. Previous studies, indeed, have reported that activity trackers can increase physical activity [85]. However, we acknowledge this potential systematic bias in our study, as done in previous work from our group [54], as our aim was to implement smartwatch in a way that closely reflected real-world conditions and use. Restricting access to the data, indeed, would have deviated from typical usage by the general population.

Finally, the use of consumer-grade smartwatches raises additional concerns, particularly regarding data security, privacy, and access to raw data. It should be noted, however, that the use of the device in our study adhered to the manufacturer’s instructions, and data storage and processing were therefore compliant with European Union regulations. A further limitation lies in the fact that raw data from Garmin devices are not accessible or downloadable through the user dashboard, which may restrict the development of more advanced and standardized analytical pipelines. Nonetheless, this was beyond the scope of our study, as our aim was to employ the smartwatches in a way that closely reflected their common use in daily life.

## Conclusions

Our findings suggest that executive functions may contribute, albeit modestly, to mobility among PwPD, especially during more demanding tasks such as backward walking. While cognitive functions show significant associations with supervised mobility, their impact on unsupervised, real-world mobility appears less pronounced. Moreover, cognitive contribution did not remain significant once clinical and demographic factors were considered, likely reflecting the relevant interplay of individual and environmental factors. Moreover, although global and executive functions can be associated with mobility, their contribution is outweighed by stronger determinants such as clinical and demographic variables. These results suggest that comprehensive PD management should integrate cognitive assessments alongside motor evaluations, emphasizing interventions that address both cognitive and environmental barriers to improve patient outcomes. Future research should further explore the interplay between cognition, mobility, and other non-motor symptoms in PD through longitudinal designs and advanced monitoring technologies, to better capture the multifaceted challenges faced by PwPD.

## Data Availability

The datasets used and/or analysed during the current study are available from the corresponding author on reasonable request.
